# Being Sick and Yet Present—The Connection Between Personality Traits and Presenteeism Behaviour Among Nurses

**DOI:** 10.1002/nop2.70584

**Published:** 2026-05-06

**Authors:** Joana Jost, Christian Eissler, Christoph Golz

**Affiliations:** ^1^ School of Health Professions Bern University of Applied Sciences Bern Switzerland; ^2^ Department of Abdomen, Inselspital Bern University Hospital, University of Bern Bern Switzerland

**Keywords:** big five, nurses, personality traits, presenteeism

## Abstract

**Aim:**

To explore the association between nurses' personality traits and their presenteeism behaviour.

**Design:**

Cross‐sectional study.

**Methods:**

Nurses from various settings in Switzerland were recruited through convenience and snowball sampling. Data was collected using an online questionnaire. A multiple linear regression was conducted for data analysis.

**Results:**

A total of 255 questionnaires were included in the analysis. Presenteeism is moderate among nurses with a mean of 2.73 (SD = 0.93). The participants were less prone to presenteeism if a physician had advised them to stay away from work. The results indicated that consciousness, neuroticism and being a nursing assistant with formal education and working in a nursing home had a significant positive correlation with nurses' presenteeism behaviour.

**Conclusion:**

Conscientiousness may indicate a sense of duty among nurses. Neuroticism could be reflected in higher emotional stress and susceptibility to stress. Workload and role demands may be associated with higher presenteeism scores among nursing assistants with formal education. Increased staffing needs in nursing homes may explain elevated presenteeism scores.

## 1 Introduction

There is a global shortage of nursing staff (World Health Organization 2020). In Switzerland, the future demand for nursing staff is expected to increase at all levels (Merçay et al. 2021). Staff retention plays a decisive role in addressing the shortage of nurses. Dealing with nurses' physical and mental stress is essential in retaining them in the profession (Merçay et al. 2021). In this context, presenteeism can represent a significant dimension (Peter et al. 2023). Presenteeism refers to employees working despite being ill (Oster and Mücklich 2019). Nursing has one of the highest rates of presenteeism (Gerlach et al. 2024; Min et al. 2022): Globally, about 49.2% of nurses are affected by presenteeism (Min et al. 2022). Poor health is a key factor in determining whether to attend work and is influenced by various personal and workplace‐related factors (Rainbow et al. 2021; Steidelmüller 2020). Personal factors include emotional exhaustion, stress, and family–work conflict (Lohaus and Habermann 2019). Workplace‐related factors are role demands, e.g., workload, the number of patients/clients and physical demands, time pressure, overtime, and overall job demands (Lohaus and Habermann 2019). An additional reason may be feelings of guilt towards patients, as nurses are responsible for those in need of care (Rainbow et al. 2021). Furthermore, pressure from managers to perform can influence the behaviour of nurses (Shan et al. 2023). Attitudes towards the workplace and colleagues, and organisational aspects such as administrative regulations or culture, may be related to the decision to presenteeism (Gerlach et al. 2024). Often, socially stigmatised illnesses, such as mental health complaints of nurses, are not considered severe enough to stay away from work (Rainbow et al. 2021).

Personality is described as an influencing factor of presenteeism (Banks and Pearson 2021). The five‐factor model, also known as the Big Five, is the predominant personality model in trait psychology and remains one of the most empirically supported models for describing individual differences (Reed 2025). It provides an integrative system, as it combines different personality systems into a single framework and integrates traits from various personality theories and models into a unified structure (Reed 2025). The five dimensions are summarised under the acronym OCEAN: Openness to new experiences, Conscientiousness, Extraversion, Agreeableness, and Neuroticism. The Big Five are determined by poles of opposites (Reed 2025).

Openness is defined as imagination, intellectual curiosity, aesthetic sensitivity, willingness to try new things, and openness to new ideas and experiences (Abu Raya et al. 2023). Open‐minded people have a wide range of interests, like to try out new things and visit new places. They tend to be curious and to require variety and novelty. Individuals who are closed tend to have fixed habits and prefer the familiar. They are more traditional and pragmatic (Abu Raya et al. 2023).

The personality trait conscientiousness describes the tendency towards self‐control, responsibility towards others, diligence, orderliness, and adherence to rules (Turner and Hodis 2025). It comprises several facets such as self‐control, orderliness, diligence, and responsibility (Turner and Hodis 2025). Conscientious people are considered reliable, responsible, compliant, disciplined, demanding, prudent, orderly, persistent and achievement oriented. People with low levels of conscientiousness are described as careless, irresponsible, lazy, impulsive, disorganised, chaotic and less achievement‐oriented (Barrick et al. 2001; Turner and Hodis 2025).

Evidence indicates that nurses tend to score high on conscientiousness, and this trait has been shown to be positively associated with safety attitudes (Kil and Chatzi 2025). Conscientiousness was also the personality trait most consistently linked to safer attitudes and behaviours across multiple occupational settings, including healthcare (Kil et al. 2024). In their quantitative study, Kil and Chatzi (2025) found that higher conscientiousness scores among nurses and midwives correlated with more favourable safety attitudes, suggesting that this trait may support adherence to procedures and error‐reducing behaviour.

Extraversion reflects a tendency towards positive affect, assertive behaviour and a preference for social interaction and attention (Angelini 2023). Characteristics of extroverts are energy, dominance, spontaneity and sociability (Angelini 2023). They tend to be talkative, sociable and assertive (Barrick et al. 2001). Individuals low in extraversion tend to be more reserved, quiet, and less socially engaged (Angelini 2023).

Agreeableness refers to individual differences in motivation to maintain positive relationships with others (Wilmot and Ones 2022). People with high scores on agreeableness are described as good‐natured, cooperative, helpful, caring, trusting, friendly and are concerned about others (Barrick et al. 2001; Wilmot and Ones 2022). Individuals with low levels of agreeableness may tend to be brusque or even rude in their interactions with others and are described as argumentative, inflexible, uncooperative, uncaring, intolerant and intransigent (Barrick et al. 2001; Hilbig and Moshagen 2025).

Neuroticism is also referred to as emotional instability and reflects a tendency to experience negative emotional states (Angarita‐Osorio et al. 2024). Individuals with high levels of neuroticism are associated with anxiety, hostility, insecurity, and depressed mood (Angarita‐Osorio et al. 2024; Barrick et al. 2001). They are less able to regulate negative emotions, show heightened stress reactivity, and tend to cope worse with stress than others (Nilsen et al. 2024). Low levels of neuroticism indicate emotional stability. These individuals are typically calm, balanced, and relaxed, and can cope with stressful situations without becoming upset.

Research also indicates that neuroticism is negatively associated with safety attitudes among nurses (Kil and Chatzi 2025). The scoping review by Kil et al. (2024) further reported that neuroticism frequently showed positive associations with unsafe or risk‐related attitudes and behaviours across multiple occupational settings, suggesting that emotional instability may amplify vulnerability in complex or high‐pressure environments. Within nursing, such tendencies could affect stress regulation, concentration, and situational awareness, which may have implications for safety‐relevant decision‐making, including working while unwell.

Nurses are typically characterised by high levels of agreeableness, cooperativeness, and interpersonal skills, which are associated with empathy and prosocial behaviour (Louwen et al. 2023). The association between personality and presenteeism has been researched in other sectors. It has been shown that the more conscientious and emotionally unstable a person is, the more prone they are to presenteeism (Jayaweera and Dayarathna 2019), which was also found for nurses (Shan et al. 2020). A previous study found, that nurses in an acute care hospital have low emotional stability, which is associated with increased presenteeism behaviour (Banks and Pearson 2021). However, they did not find an association between conscientiousness and presenteeism (Banks and Pearson 2021), which raises the question of which results now apply to nurses regarding the association between personality traits and presenteeism.

Therefore, the study aimed to elaborate on the relationship between personality traits and presenteeism among nurses, considering other socio‐demographic characteristics.

## 2 Method

### 2.1 Design

This study is based on a cross‐sectional study design. We adhered to the STROBE reporting guideline for cross‐sectional studies (Von Elm et al. 2007).

### 2.2 Recruitment and Study Sample

A convenience sampling among nurses from the German‐speaking part of Switzerland was conducted. The study participants were selected via the author's extended professional network. Potential participants were contacted by email or messaging platforms and informed about the research project. The detailed study information and the link to the online questionnaire were included in the email or message. The convenience sampling was supplemented with a snowball sampling. Participants were asked to forward the email to acquaintances.

Nurses from all settings (acute and rehabilitation settings, psychiatric settings, long‐term settings and hospital‐external care) living and working in Switzerland were included. Nurses with different levels of education and professional positions, as well as nurses in training were also included. Nurses, who were unable to complete a questionnaire in German were excluded. The inclusion criteria were checked based on the socio‐demographic information provided when completing the questionnaire.

### 2.3 Data Collection

Data was collected between October and December 2023. The online questionnaire was conducted using LimeSurvey. Before data collection, the self‐developed socio‐demographic items and introductory text were tested for clarity using the think‐aloud method (Noushad et al. 2024) with nine nurses from the author's circle of acquaintances, representing different educational levels. Based on this feedback, the introductory text was revised by adding a definition of presenteeism. Presenteeism was defined as working despite physical or psychological health impairments when there is a reason to stay away from work so that participants could clearly understand the concept. The nine nurses also participated in a pretest of the online questionnaire for potential errors.

### 2.4 Measurement

The questionnaire consisted of socio‐demographic information (e.g., gender, age, origin, professional function, etc.), the German version Hägerbäumer presenteeism scale (Hägerbäumer 2017) to measure presenteeism and the German Big Five Inventory (BFI‐K) (Rammstedt and John 2005) to measure personality traits.

The Hägerbäumer scale is comprised of six items (Hägerbäumer 2017). The answers are recorded on a six‐point Likert scale from 1 *never when I was ill* to 5 *very often when I was ill*. The sixth answer option is defined as 0 *I was not ill*. The presenteeism score is calculated from the mean values of the six items. The Hägerbäumer scale is available in German and is classified as a reliable and valid instrument for assessing presenteeism (*α* = 0.89) (Hägerbäumer 2017). Internal consistency for the presenteeism scale in the present sample was also high (Cronbach's *α* = 0.89).

To assess personality, the short version of the Big Five Inventory (BFI‐K) (Rammstedt and John 2005) was used. The Big Five Inventory (BFI) (John et al. 1991) was developed to measure the Big Five prototypical five factors of personality. The BFI‐K measures the dimensions of extraversion, agreeableness, conscientiousness and neuroticism with four items each. The openness to experience dimension is represented by five items. The response options are represented using a five‐point Likert scale from *very inapplicable* to *very applicable*. The BFI‐K is a brief and widely used instrument with established validity and acceptable internal consistencies across its subscales (*α* = 0.59–0.86) (Rammstedt and John 2005). In the present sample, internal consistency ranged similarly from *α* = 0.59 to 0.80 across the five personality dimensions. While reliability was acceptable for most scales, the lower value for conscientiousness is consistent with the short scale length of the BFI‐K.

### 2.5 Data Analysis

Data was analysed using R 2023.9.1.494 (Posit Team 2023). The questionnaire was completed anonymously. A plausibility check was carried out. Implausible data and incomplete data sets were excluded. If the data did not match the inclusion criteria, the questionnaire was excluded. Nurses, who were not ill during the last 12 months were excluded, because presenteeism was not indicated due to not being ill. The data on socio‐demographic characteristics and personality were analysed using descriptive statistics. The arithmetic mean and the standard deviation (SD) were calculated.

The assumptions for multiple linear regression (interval scaling, linearity of the correlation, normal distribution of the residuals, homoscedasticity and multicollinearity) were checked a priori. In case of missings, we computed listwise deletion. Participants who reported that they had not been ill for the last 12 months were excluded from the mean score calculation of the Hägerbäumer Presenteeism scale (Hägerbäumer 2017). Presenteeism was defined as the dependent variable. Socio‐demographic data and the five personality traits were defined as independent. *p*‐values (*p*) below 0.05 and confidence intervals (CI) of 95% were considered significant.

### 2.6 Ethical Approval & Consent to Participate

The local Swiss ethical board in Bern confirmed that the study does not warrant a full ethical application and does not fall under the Swiss Federal Act on Research Involving Human Beings (Req‐2023‐01098). The study was conducted on a voluntary basis for all participants were free to stop filling in the questionnaire at any time. Participants received written information before the start of the study about the contents, aim, and voluntary nature of their participation and gave their informed consent by completing the first survey page. All participants were able to interrupt the questionnaire at any time without any explanation. All procedures followed were in accordance with the ethical standards of the responsible committee on human experimentation (institutional and national) and with the Helsinki Declaration of 1975, as revised in 2000. Informed consent was obtained from all patients for being included in the study.

## 3 Results

### 3.1 Response Rate

**FIGURE 1:**
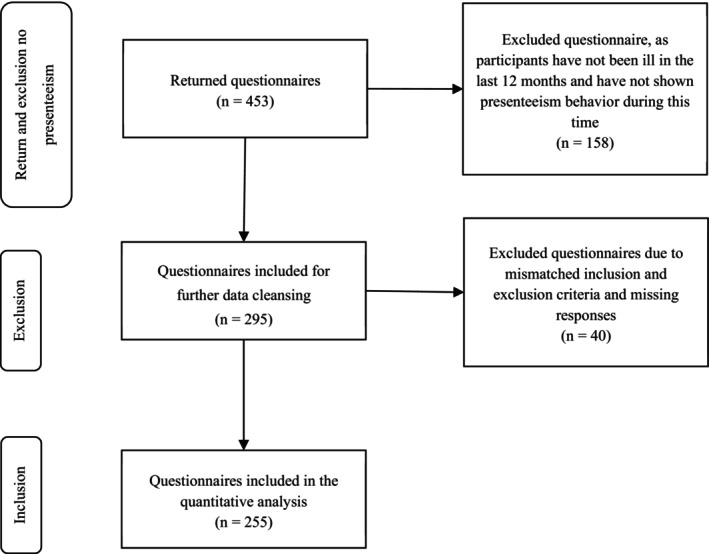
Representation of the sample.

A total of 453 people answered the online questionnaire. 158 participants who had not been ill in the past 12 months were excluded as they did not exhibit presenteeism behaviour during this time. Due to missing information or mismatched inclusion and exclusion criteria, 40 questionnaires were excluded from the data analysis (Figure 1). As an example, *n* = 1 was working in a school setting and *n* = 2 were working in an outpatient setting, which may refer to either a day hospital or a general practitioner's practice and no clear allocation was possible.

### 3.2 Participant Characteristics

Of the 255 participants, 91.4% were female (*n* = 233) and the average age of the participants was 32.96 years (SD = 9.86). 91.4% of the participants were born in Switzerland (*n* = 233). Most participants were nurses with a bachelor's degree in nursing (*n* = 105, 41.2%). Most of the participants worked in acute care hospitals (*n* = 189, 74.1%), with the majority (*n* = 81, 42.9%) being nurses with a bachelor's degree (see Table 1).

**TABLE 1:** Description of the sample.

Feature	*n* (%)	Mean (SD)
Gender		
Female	233 (91.4)	
Male	22 (8.6)	
Age in years		32.96 (9.86)
Origin		
Switzerland	233 (91.4)	
Other	22 (8.6)	
Profession		
APN, NP, CNS	33 (12.9)	
Registered Nurses with a bachelor's degree	105 (41.2)	
Registered Nurses without a bachelor's degree	94 (36.9)	
Nursing assistants with formal education	16 (6.3)	
Nursing aides	7 (2.7)	
In training for the specified profession		
No	182 (71.4)	
Yes	73 (28.6)	
Completed further training		
No	149 (58.4)	
Yes	106 (41.6)	
Management position		
No	186 (72.9)	
Yes, without personnel responsibility	41 (16.1)	
Yes, with personnel responsibility	28 (11.0)	
Care of own family members (children, parents, etc.)		
No	187 (73.3)	
Yes	68 (26.7)	
Full‐time equivalent in percent (%)		76.94 (25.04)
Setting		
Acute hospital	189 (74.1)	
Nursing home	18 (7.1)	
Psychiatry	12 (4.7)	
Rehabilitation and geriatrics	5 (2.0)	
Hospital‐external care	31 (12.2)	
Total experience in nursing care in years		11.90 (8.81)

*Note:* Sample size *n* = 255.

#### 3.2.1 Presenteeism

The average presenteeism score was 2.73 (SD = 0.93). Table 2 shows the individual mean values of the items, which ranged from 1.63 (item 2, SD = 1.08) to 3.33 (item 5, SD = 1.25). It can be seen that the participants were less prone to presenteeism if a physician had advised them to stay away from work (item 2). The Hägerbäumer ranges on a six‐point Likert scale ranging from *1 never when I was ill to 5 very often when I was ill* (Hägerbäumer 2017).

**TABLE 2:** Description of the sample according to presenteeism behaviour.

Presenteeism	Mean (SD)
Presenteeism Overall	2.73 (0.93)
*Item 1: I came to work despite being ill*	3.14 (1.05)
*Item 2: I worked even though my doctor advised me not to*	1.63 (1.08)
*Item 3: I worked despite severe symptoms of illness (e.g., pain, chills, fever)*	2.26 (1.18)
*Item 4: I worked the full working day or shift despite being ill*	3.01 (1.22)
*Item 5: I have taken medication due to acute complaints in order to be able to work*	3.33 (1.25)
*Item 6: Although I was ill, I dragged myself to work*	3.02 (1.13)

*Note:* Sample size *n* = 255. Presenteeism items: Scale from *1 never to 5 very often. Overall presenteeism* is calculated from the mean values of the individual items.

### 3.3 Personality Traits

The mean values of the personality traits were between 2.79 (neuroticism, SD = 0.77) and 4.14 (conscientiousness, SD = 0.53). The average value in the openness to experience dimension was 3.71 (SD = 0.68). The other values can be found in Table 3.

**TABLE 3:** Description of the sample according to personality traits.

Personality traits	Mean (SD)
Extraversion	3.63 (0.81)
Compatibility	3.72 (0.73)
Conscientiousness	4.14 (0.53)
Neuroticism	2.79 (0.77)
Openness to experience	3.71 (0.68)

*Note:* Sample size *n* = 255. Characterisation of personality traits: Scale from 1 *very inapplicable* to 5 *very applicable*.

#### 3.3.1 Results of the Multiple Linear Regression Model for Presenteeism

The regression model explains 13.47% of the variance. Table 4 summarises the results of the multiple linear regression model for presenteeism. The model explained 21% of the variance: *R* ^2^ = 0.21, the overall *F*‐test resulted in *F*(23, 231) = 2.72, *p* < 0.001. Table 4 shows that the dimension conscientiousness has a significant positive correlation with presenteeism (*β* = 0.335, 95%‐CI [0.104; 0.566]). Neuroticism shows a positively significant correlation with presenteeism (*β* = 0.168, 95%‐CI [0.002; 0.333]). The other personality traits, on the other hand, show no significant correlation with presenteeism behaviour. Regarding the occupational groups, healthcare assistants show a significant positive increase in presenteeism (*β* = 0.758, 95%‐CI [0.272; 1.245]) compared to nurses with a bachelor's degree. Nurses from nursing homes (*β* = 0.475, 95%‐CI [0.035; 0.914]) show significantly higher average presenteeism behaviour compared to nurses in acute care hospitals (intercept) (*β* = 1.182, 95%‐CI [−0.525; 2.889]).

**TABLE 4:** Multiple linear Regression.

Factors	Beta *β*	Std. error	*t*‐value	*p*‐value	95%‐CI
Intercept	1.18	0.87	1.36	0.17	−0.52 to 2.89
Male	0.17	0.21	0.77	0.44	−0.26 to 0.59
Age in years	−0.02	0.01	−1.37	0.17	−0.05 to 0.01
Other origin	0.17	0.21	0.82	0.41	−0.25 to 0.59
Profession	0.17	0.19	0.86	0.39	−0.21 to 0.55
APN, NP, CNS					
Profession	0.19	0.14	1.33	0.18	−0.09 to 0.47
Registered Nurses without a bachelor's degree					
Profession	0.76	0.25	3.07	**0.00**	0.27 to 1.24
Nursing assistants with formal education					
Profession	0.71	0.37	1.91	0.06	−0.02 to 1.44
Nursing aides					
In training for the specified profession	−0.11	0.14	−0.78	0.44	−0.39 to 0.17
Completed further training	0.07	0.14	0.54	0.59	−0.20 to 0.34
Management position	0.06	0.17	0.37	0.71	−0.27 to 0.40
Yes, without personnel responsibility					
Management position	−0.09	0.19	−0.47	0.64	−0.47 to 0.29
Yes, with personnel responsibility					
Care of own family members (children, parents, etc.)	0.22	0.15	1.51	0.13	−0.07 to 0.52
Full‐time equivalent in percent (%)	0.00	0.00	0.12	0.90	0.00 to 0.01
Setting	0.47	0.22	2.13	**0.03**	0.03 to 0.91
Nursing home					
Setting	−0.20	0.27	−0.75	0.46	−0.75 to 0.34
Psychiatry					
Setting	0.24	0.41	0.59	0.56	−0.57 to 1.05
Rehabilitation and geriatrics					
Setting	−0.04	0.18	−0.24	0.81	−0.40 to 0.31
Spitex					
Total experience in nursing care in years	−0.02	0.02	−1.06	0.29	−0.05 to 0.01
Extraversion	0.08	0.08	1.00	0.32	−0.08 to 0.24
Compatibility	−0.12	0.08	−1.58	0.12	−0.28 to 0.03
Conscientiousness	0.34	0.12	2.86	**0.00**	0.10 to 0.57
Neuroticism	0.17	0.08	2.00	**0.05**	0.00 to 0.33
Openness to experience	0.11	0.09	1.23	0.22	−0.07 to 0.28

*Note:* Sample size *n* = 255. Presenteeism: Scale from 1 *never* to 5 *very often*. Characteristics of personality traits (extraversion, agreeableness, conscientiousness, neuroticism and openness to experience): Scale from 1 *very inapplicable* to 5 *very applicable*. Significant values are printed in bold at a significance level of 0.05; *R* ^2^: 0.2131 / *R* ^2^ adapted: 0.1347.

## 4 Discussion

The aim of this study was to show the association between presenteeism and personality traits among nurses, considering socio‐demographic data in the German‐speaking part of Switzerland. Among the participants, conscientiousness is the most pronounced and neuroticism the least. Thus, nurses seem to have rather a tendency towards self‐control, responsibility towards others, diligence, orderliness and adherence to rules. For example, a sense of duty is cited as one of the most common reasons for presenteeism among nurses (Peter et al. 2023). Further, conscientious employees feel committed to the organisation (Watzka 2021). In particular, conscientious individuals were found to have higher levels of professional commitment (Wang et al. 2024). On the other hand, nurses seem to be rather emotional stable and able to cope with stressful situation, which was also found in other studies (Wan et al. 2019). Thus, nurse managers need to be aware of the dominant presence of conscious employees in their teams and consider the potential risk for presenteeism in this context. On the other side, although coping with stressful situations may seem a strength among nurses, the research indicating high stress and long‐term consequences such as burnout among nurses (Peter et al. 2024) should be indicative for managers to invest in improving the working conditions.

In our study, behaviour presenteeism is moderate and comparable with findings from a survey conducted with nurses in Germany with mean presenteeism scores of 2.73 in our sample versus 2.65 in the previous study using the same scale (Hägerbäumer 2017). Conscientiousness correlates positively with presenteeism among nurses: the higher the values for conscientiousness, the more prone nurses are to presenteeism, which is also found in other industries (Jayaweera and Dayarathna 2019). There was also a significant positive association between neuroticism and presenteeism. This relationship has been consistently observed across occupational settings and is supported by meta‐analytic and empirical evidence (Banks and Pearson 2021; Jayaweera and Dayarathna 2019). People with high levels of neuroticism can be anxious, irritable, depressed and prone to stress (Angarita‐Osorio et al. 2024). Further, neuroticism predicts rapid subjective overload in working life (Nilsen et al. 2024). For nurses in hospital settings, high levels of neuroticism are associated with high work‐related stress, which is considered a predictor of presenteeism (Banks and Pearson 2021). Neuroticism is also negatively associated with safety attitudes, suggesting that emotionally unstable individuals may engage less consistently in safety‐related behaviours (Kil and Chatzi 2025). Considering that presenteeism has been associated with impaired cognitive functioning, reduced decision quality and increased risk of errors in clinical practice, the interplay between personality traits, presenteeism, and safety‐relevant behaviour warrants further investigation. Future studies should therefore examine whether personality‐based vulnerability to presenteeism may also translate into patient safety risks, for example by integrating both presenteeism and safety‐attitude outcomes or linking behavioural data with clinical performance indicators. The remaining personality traits were not significantly related to presenteeism in this study and other studies report different findings. For example, Banks and Pearson (2021) found higher levels of extraversion and openness among nurses associated with a higher level of presenteeism. In other industries, however, extraversion and openness are negatively correlated with presenteeism (Jayaweera and Dayarathna 2019). The extent to which industry‐specific conditions account for these divergent findings is not yet sufficiently understood and warrants further empirical examination, ideally through comparative, sector‐specific designs that consider contextual work characteristics, norms, and exposure to health risks.

Therefore, organisational measures should start at different points to match the personality traits relevant for presenteeism behaviour. Rules at work should be adapted to discourage or minimise presenteeism behaviour. One proposed measure is granting paid sick leave, as organisational policies and top‐down strategies play a key role in shaping presenteeism behaviour (Nowrouzi‐Kia et al. 2025). However, in Switzerland, this is already mandatory by law and, therefore, not the most promising measure to mitigate presenteeism behaviour. It rather seems relevant to target the implicit rules, respectively the responsibility towards others by such as the feeling of not being able to leave the team alone in times of workforce shortage, which is done with a health‐promoting collaboration (Komp et al. 2022). Regarding neuroticism, targeted support services should be provided for nurses with higher levels of emotional instability, focusing on coping strategies. This may include workplace wellbeing programs and awareness‐raising measures, as organisational interventions and mental health support have been identified as key approaches to addressing presenteeism (Nowrouzi‐Kia et al. 2025). The resources of relaxation, mindfulness and work‐life balance could be strengthened through training, as they can mitigate the negative health effects of presenteeism (Komp et al. 2021).

In this study, nurses in nursing homes had significantly higher presenteeism scores than in other settings. This was also found in a previous study in Switzerland (Peter et al. 2023). This result was explained by the higher degree of understaffing in long‐term care. In Swiss nursing homes, the need for nursing staff will be just under 50% by 2035. By comparison, the requirement in acute hospitals is 23% by 2035 (Merçay et al. 2021). In another study was shown, that 26.3% of nurses in nursing homes exhibited presenteeism within one month. A positive correlation was found between presenteeism and increased rationing of activities of daily living by residents (Dhaini et al. 2017). The rationing of activities of daily living was associated with other physical symptoms and emotional exhaustion. It was recommended that targeted measures be taken to promote nurses' health, including monitoring emotional distress and associated symptoms. However, appropriate measures must be developed and tested (Dhaini et al. 2017). The high workload in nursing homes can also be an explanation for presenteeism behaviour. The workload and the number of patients to be cared for are cited as factors influencing presenteeism (Lohaus and Habermann 2019). Further research is needed to determine whether reducing workload, improving staffing ratios, or redistributing responsibilities can effectively prevent presenteeism in long‐term care settings.

### 4.1 Strengths and Limitations

This study has several strengths. These include relevance of content, methodological and ethical rigour as well as adherence to the STROBE reporting guidelines. The research question is derived from the current state of research. Reliable and valid instruments were used for operationalisation (Hägerbäumer 2017; Rammstedt and John 2005). Furthermore, data quality was ensured by carefully collecting the data using an online questionnaire. A pre‐test was carried out as a matter of priority in order to eliminate any technical, organisational or time‐related difficulties. In addition, the questions were checked for comprehensibility.

Despite these strengths, the study has some limitations that should be considered. Due to the cross‐sectional design, no causal conclusions can be drawn, and the convenience sampling is not representative. Furthermore 158 people stated that they had not been ill in the last 12 months. This corresponds to 34.8%. It could be a case of memory bias if the participants could no longer remember feeling ill due to e.g., headaches or mental health problems. Additionally, the factors included in the model explain 13.47% of the variation in the dependent variable. The model only explains a limited part of the variation. Important factors are missing or there may be other factors influencing the variation that are not included in the model. The use of the Big Five personality model must be viewed critically, as the model is not based on an explicit theory of development and was developed purely through linguistic analysis. The lexical approach on which the Big Five is based must also be critically scrutinised. There are doubts as to whether individual adjectives are sufficient to describe the complexity of personality.

## 5 Conclusions

This study found that presenteeism among nurses is moderate, with conscientiousness and neuroticism being positively associated with presenteeism. These findings contribute to a better understanding of individual factors associated with presenteeism in nursing, and they may serve as a basis for identifying risk profiles for preventive interventions. Future studies could complement these results by examining causal pathways, moderating factors and mechanisms, as well as the interaction between personality traits, workplace conditions and subjective illness perception.

The relationship between personality and presenteeism should be investigated further, taking into account known influencing factors. In order to investigate the phenomenon of subjective illness perception and the resulting differences in presenteeism behaviour in more detail, a group comparison would be useful in further research. The difference between physical and psychological symptoms in relation to presenteeism could also be investigated in further studies. The relationship between dealing with stress and presenteeism as well as the relationship between presenteeism and quality of care could also be part of future studies. In addition, personality traits in connection with training and industry differences in relation to presenteeism behaviour should be examined more closely. With a larger and representative sample, the differences within the occupational groups could also be examined in more detail.

Apprentices and students can be sensitised to the topic of presenteeism and its consequences during their training and education. Their own awareness can help future nurses to better understand their own behavioural patterns. The focus can also be placed on self‐reflection to recognise one's own personality and tendency towards presenteeism. The importance of self‐care can be highlighted, and learners should be encouraged to take their health and well‐being seriously.

Employers and managers in the healthcare sector should be aware of the role of personality traits, in particular conscientiousness and neuroticism, in view of the risk of presenteeism. Working conditions should be adapted so that, for example, a replacement is available for the missing person. Organisational culture is required to recognise the importance of self‐care. Incentives and programs for health‐conscious behaviour can be created. Raising awareness of presenteeism and a clear attitude that presenteeism is undesirable in the workplace can also contribute to prevention.

## Author Contributions

J.J. C.E. and C.G. contributed to the study conception and design. Material preparation, data collection, and analysis were performed by J.J. The first draft of the manuscript was written by J.J. C.E. and C.G. commented on the previous versions of the manuscript. All authors read and approved the final manuscript.

## Funding

The authors have nothing to report.

## Consent

Participants received written information before the start of the study about the contents, aim, and voluntary nature of their participation and gave their informed consent by completing the first survey page. All participants were able to interrupt the questionnaire at any time without any explanation. All procedures followed were in accordance with the ethical standards of the responsible committee on human experimentation (institutional and national) and with the Helsinki Declaration of 1975, as revised in 2000. Informed consent was obtained from all patients for being included in the study.

## Conflicts of Interest

The authors declare no conflicts of interest.

## Data Availability

The data that support the findings of this study are available from the corresponding author upon reasonable request.
